# Sepsis-coded hospitalisations and associated costs in Australia: a retrospective analysis

**DOI:** 10.1186/s12913-023-10223-1

**Published:** 2023-11-29

**Authors:** Ashwani Kumar, Naomi Hammond, Brett Abbenbroek, Kelly Thompson, Colman Taylor, Bala Venkatesh, Anthony Delaney, Simon Finfer

**Affiliations:** 1 Critical Care Program, The George Institute for Global Health, Sydney, NSW Australia; 2 Royal North Shore Hospital, Sydney, NSW Australia; 3 Nepean Blue Mountains LHD, Sydney, NSW Australia; 4 School of Public Health, Imperial College London, London, UK; 5 Princess Alexandra Hospital, Brisbane, QLD Australia; 6 University of New South Wales, Sydney, Australia

**Keywords:** Sepsis, Incidence, Trends, ICD codes, Australian-Refined diagnosis related Group

## Abstract

**Objective:**

To report trends in Australian hospitalisations coded for sepsis and their associated costs.

**Design:**

Retrospective analysis of Australian national hospitalisation data from 2002 to 2021.

**Methods:**

Sepsis-coded hospitalisations were identified using the Global Burden of Disease study sepsis-specific ICD-10 codes modified for Australia. Costs were calculated using Australian-Refined Diagnosis Related Group codes and National Hospital Cost Data Collection.

**Results:**

Sepsis-coded hospitalisations increased from 36,628 in 2002-03 to 131,826 in 2020-21, an annual rate of 7.8%. Principal admission diagnosis codes contributed 13,843 (37.8%) in 2002-03 and 44,186 (33.5%) in 2020-21; secondary diagnosis codes contributed 22,785 (62.2%) in 2002-03 and 87,640 (66.5%) in 2020-21. *Unspecified sepsis* was the most common sepsis code, increasing from 15,178 hospitalisations in 2002-03 to 68,910 in 2020-21. The population-based incidence of sepsis-coded hospitalisations increased from 18.6 to 10,000 population (2002-03) to 51.3 per 10,000 (2021-21); representing an increase from 55.1 to 10,000 hospitalisations in 2002-03 to 111.4 in 2020-21. Sepsis-coded hospitalisations occurred more commonly in the elderly; those aged 65 years or above accounting for 20,573 (55.6%) sepsis-coded hospitalisations in 2002-03 and 86,135 (65.3%) in 2020-21. The cost of sepsis-coded hospitalisations increased at an annual rate of 20.6%, from AUD199M (€127 M) in financial year 2012 to AUD711M (€455 M) in 2019.

**Conclusion:**

Hospitalisations coded for sepsis and associated costs increased significantly from 2002 to 2021 and from 2012 to 2019, respectively.

**Supplementary Information:**

The online version contains supplementary material available at 10.1186/s12913-023-10223-1.

## Introduction

Sepsis is defined as life-threatening organ dysfunction due to a dysregulated immune response to infection [1]. In 2017, the World Health Organisation (WHO) recognised sepsis as a global health priority [2], and the Global Burden of Disease (GBD) project estimated that in the same year almost 50 million sepsis cases and 11 million sepsis-related deaths occurred worldwide [3]. The GBD estimate for Australia in 2017 was 55,217 cases and 8,700 deaths, 5.4% of Australian deaths that year.

Obtaining accurate population-based estimates for sepsis epidemiology is challenging due to the absence of a definitive diagnostic test. Most large scale epidemiological studies use the WHO’s International Classification of Diseases (ICD) [4] to estimate sepsis incidence and trends [5, 6]. Although considerable variation in sepsis estimates are reported based on different ICD coding methods, the number of ICD codes and their version, source of data and reference standard, they are currently the only practical way to study trends in sepsis incidence at the global, regional and national level [7].

Although other countries have reported an increasing incidence of sepsis [8–10], we know little about trends in sepsis hospitalisations in Australia. A 2014 report estimated mortality rates for patients with sepsis admitted to Australian ICUs that reported data to the Australian and New Zealand Intensive Care Society Centre for Outcome and Resource Evaluation (ANZICS CORE) [11]. As reporting to ANZICS CORE is voluntary and does not include all Australian ICUs or cases occurring outside of ICU, those data give an incomplete picture of sepsis in Australia. In 2020, the GBD project estimated the global incidence of sepsis between 1990 and 2017 using death certificate data and ICD codes to indirectly derive incidence by estimating case fatality rates [3]. For individual countries, including Australia, it only provided an incidence estimate for 2017.

Internationally sepsis imposes a significant financial burden on healthcare systems. Sepsis is ranked as the most expensive clinical condition in the United States with an annual cost of USD38.2 billion (€35.9 billion) in 2017 [12], while in Australia the total annual cost of sepsis is estimated to be between AUD1.5 billion (€0.97 billion) and 4.8 billion (€3.1 billion) [13]. However, no study has reported temporal trend of costs associated with sepsis hospitalisations in Australia.

To address these knowledge gaps, we estimated national-level trends in sepsis-coded hospitalisations in Australia between 2002 and 2021 and associated costs between 2012 and 2019.

## Methods

This was a retrospective analysis of a national aggregated dataset provided by the Australian Institute of Health and Welfare (AIHW). As we used aggregated administrative datasets the need for ethical approval was waived by local regulations.

### Data source

In Australia, all hospitalisations are assigned diagnostic and procedural codes; International Statistical Classification of Diseases and Related Health Problems, Tenth Revision, Australian Modification (ICD-10-AM) codes by clinical coders and then grouped into Australia Refined Diagnosis-Related Groups (AR-DRGs) to record health conditions and resource use according to Australian Coding Standards [14].

Australian states report ICD-10-AM codes and AR-DRGs for all hospitalised patients to the National Hospital Admission Data Collection. This dataset contains aggregated hospital admission data on patients admitted to all public and private hospitals and is maintained by the AIHW. For this study, we used this aggregated dataset which includes principal and secondary diagnosis ICD-10-AM codes [15] and AR-DRGs [16]. The estimated resident population data for each corresponding year was retrieved from the Australian Bureau of Statistics [17].

The estimated costs associated with sepsis-coded hospitalisations were obtained using National Hospital Cost Data Collection (NHCDC) of the Independent Hospital and Aged Care Pricing Authority (formerly known as Independent Hospital Pricing Authority) [18]. The NHCDC contains data on health system costs and a mean length of stay to produce annual cost weights for each AR-DRG and a National Efficient Price which is based on the average cost of public hospital activity in a given year. Between 2002 and 2014–15, two sepsis AR-DRGs, Septicaemia with catastrophic complications and comorbidities (T60A) and Septicaemia without catastrophic complications and comorbidities (T60B) were in use. In 2015–16 a third sepsis AR-DRG, Septicaemia, Minor Complexity (T60C) was introduced and T60A and T60B were renamed as *Septicaemia, Major Complexity* and *Septicaemia, Intermediate Complexity*, respectively (see Supplementary file 1).

### Time period for analysis

We assessed sepsis-coded hospitalisations between 01 and 2002 to 30 June 2021. Data for associated costs were available and assessed for financial years 2012 to 2019.

### Sepsis-coded hospitalisations identification

We identified sepsis-coded hospitalisations for each year by retrieving the number of separations (principal and secondary) for each sepsis-specific ICD-10-AM code included in an *explicit* method used in a recent Australian study [19] (see Supplementary file 2 for list of codes), these codes were those used in the Global Burden of Disease study modified for the Australian context. ICD codes specific to newborn sepsis were excluded from the analysis as the focus of this study was on adult and paediatric (> 1 year of age) sepsis-coded hospitalisations.

### Data analysis

The total number of sepsis-coded hospitalisations were calculated by adding all reported sepsis-coded hospitalisation types in a year. The sepsis-coded hospitalisation incidence was calculated as follows:

*Population-level sepsis-coded hospitalisation incidence = (Total number of sepsis-coded hospitalisations/ Estimated Resident Population) x 10,000*.

*Sepsis-coded hospitalisation incidence = (Total number of sepsis-coded hospitalisations/ total number of hospitalisations) x 10,000*.

*Age-distributed sepsis-coded hospitalisation incidence = (Total number of sepsis-coded hospitalisations in a specific age group/ Estimated Resident Population) x 10,000*.

Population estimates were taken from June each year to be consistent with the reporting cycle of the National Hospital Admission Data Collection.

The proportion of ICD-10-AM codes for the most common sepsis-coded hospitalisation types and their population-level incidence was also calculated for the study period.

The associated cost for sepsis-coded hospitalisations in a given year were calculated as follows:

*Annual sepsis-coded hospitalisation associated cost (AUD) = (Number of sepsis AR-DRGs x Average cost per sepsis AR-DRG) + … (Number of sepsis AR-DRGn x Average cost per sepsis AR-DRGn)*.

### Subgroups

Sepsis-coded hospitalisations were calculated in following age groups: paediatric (1–14 years), young adult (15–39 years), middle-aged (40–64 years), elderly (65 years and older). Additionally, we compared sepsis-coded hospitalisations and their population-based incidence in the following subgroups pairs: males versus females, age 65 and older versus younger, males aged aged 65 and older versus females of same age.

### Statistical analysis

A descriptive data analysis was undertaken using Microsoft Excel with trends in sepsis-coded hospitalisation were calculated using a generalised linear regression model of Joinpoint, version 4.8.0.1 (National Cancer Institute, Bethesda, MD, USA) and reported as average annual percentage change along with 95% confidence intervals (CI). A constant variance was assumed, and autocorrelation and adjustment for seasonal effects was not needed as data were compared by calendar year. Annual changes in incidence in subgroup pairs were compared using linear regression with SPSS statistical software, (IBM Corp. Released 2020. IBM SPSS Statistics for Windows, Version 27.0. Armonk, NY: IBM Corp). Statistical significance was set at p < 0.05 with no adjustment for multiple comparisons.

## Results

### Sepsis-coded hospitalisations

The total number of hospitalisations coded as sepsis increased from 36,628 in 2002–03 to 131,826 in 2020–21 with a mean increase of 7.8% per year.

**Fig. 1:**
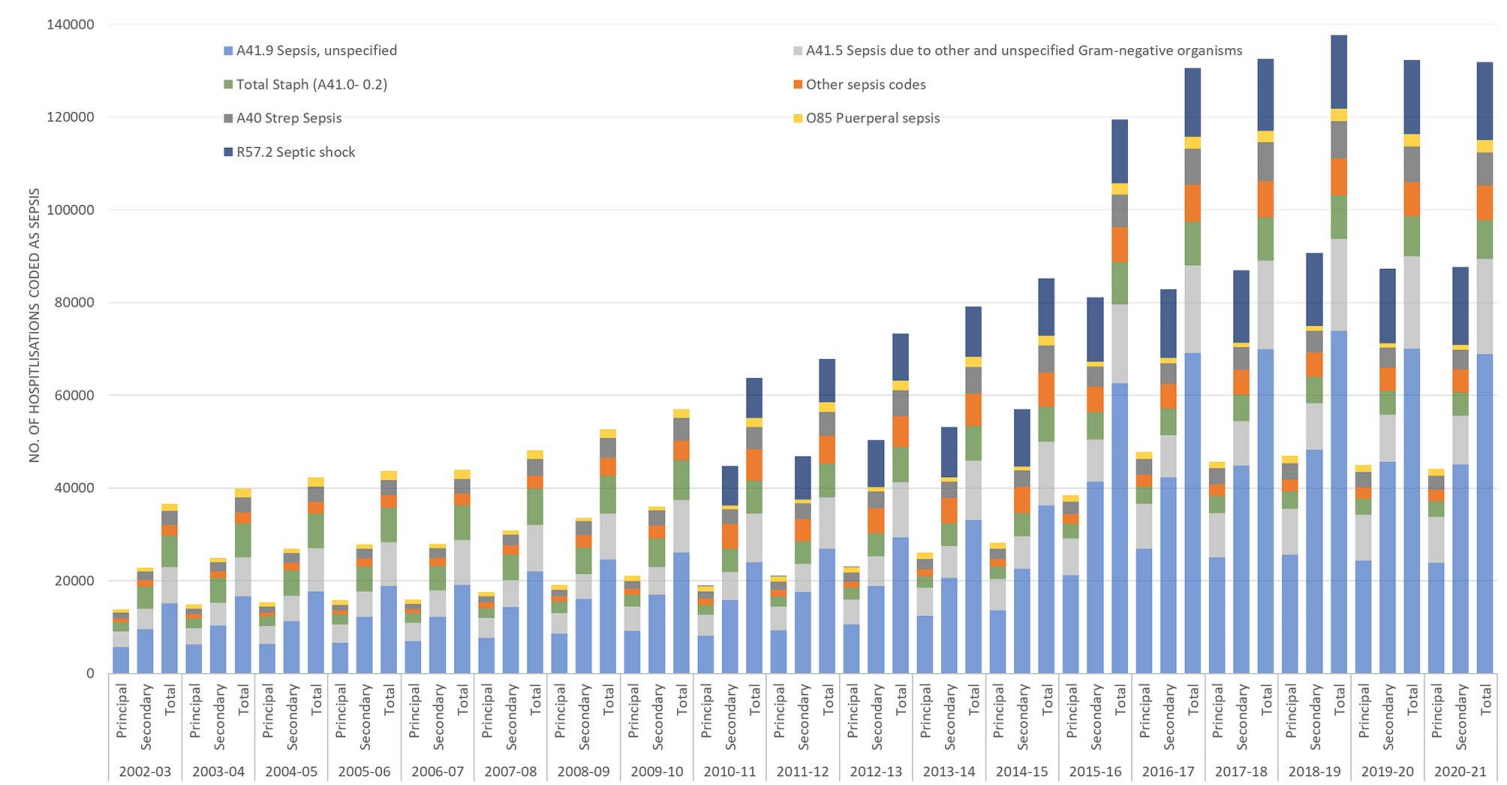
Sepsis-coded hospitalisations by sepsis ICD-10-AM codes. Note: Hospitalisations coded as other sepsis codes were calculated by adding hospitalisations coded as sepsis ICD-10-AM codes other than the top six sepsis ICD-10-AM codes displayed in the graph; ICD-10-AM: International Classification of Diseases Australian-modification

Sepsis codes within principal diagnosis codes increased from 13,843 in 2002-03 to 44,186 in 2020-21 (an increase of 219%), whereas secondary diagnoses codes increased from 22,785 in 2002-03 to 87,640 in 2020-21 (an increase of 256%). (Fig. 1 and Supplementary file 3; Table S1) The most common sepsis code was “unspecified sepsis” (A41.9), accounting for 41.4% of the total sepsis-coded hospitalisations in 2002-03 and 52.3% in 2020-21. The number of hospitalisations coded as unspecified sepsis, increased from 15,178 in 2002-03 to 68,910 in 2020-21. Hospitalisations coded as septic shock (R57.2) increased from 8,859 in 2010-11 to 16,777 in 2020-21 whereas SIRS of infectious origin with organ failure (R65.1) reduced from 2,620 to 594 during same time period. Both these codes were recorded from 2010 to 11 onwards only and all were secondary diagnosis codes. (Fig. 1 and Supplementary file 3; Table S1)

**Fig. 2:**
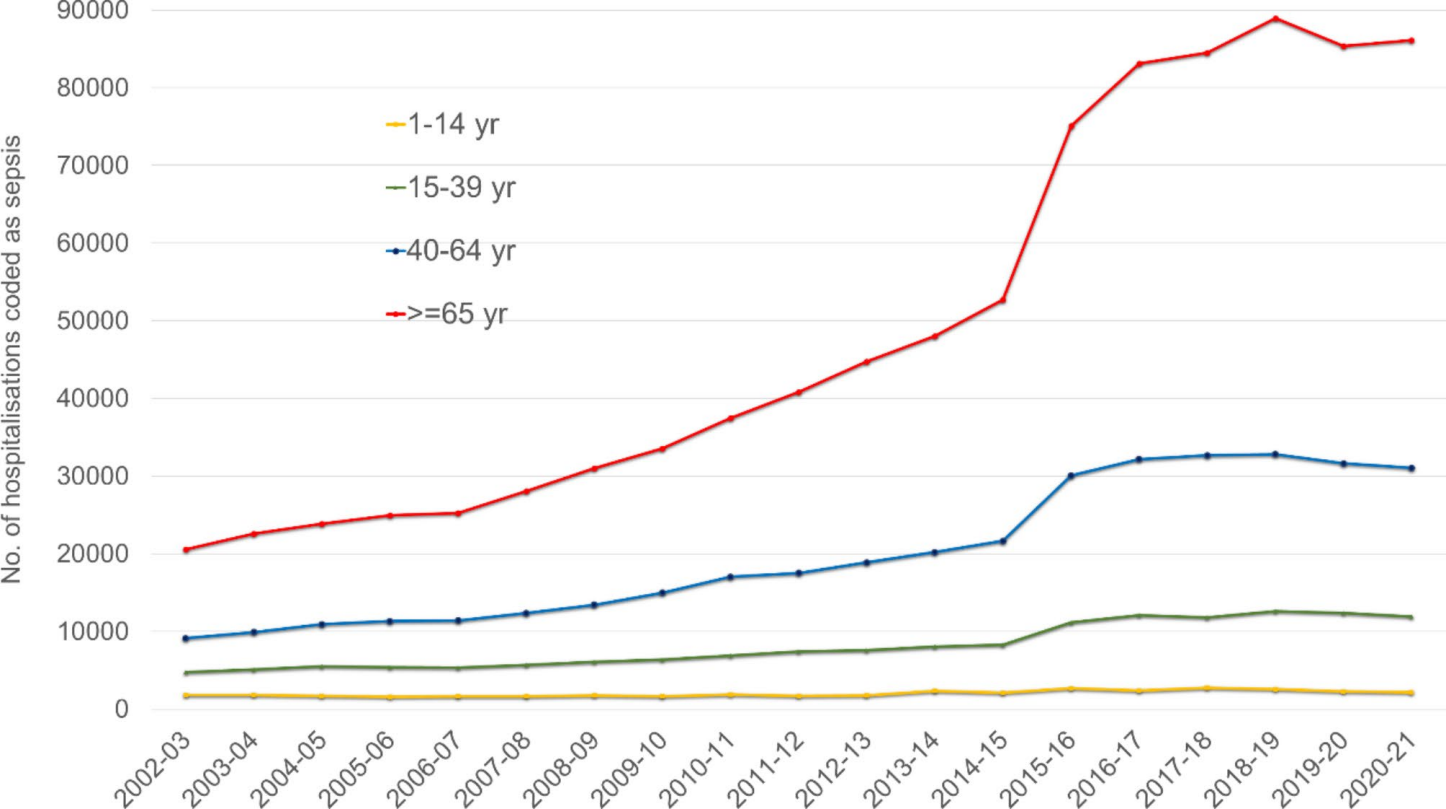
Total number of annual sepsis-coded hospitalisations by age

Sepsis-coded hospitalisations by age groups are shown in Fig. 2. Sepsis-coded hospitalisations in those aged 65 years and above increased from 20,573 in 2002-03 to 86,135 in 2020-21, an increase of 318.7%. Amongst other age groups, sepsis-coded hospitalisations increased from 9144 to 31,060 (increase of 239.7%), 4750 to 11,900 (increase of 150.5%) and 1827 to 2188 (increase of 19.8%) in those aged between 40 and 64 years, 15 to 39 years and 1 to 14 years respectively.

### Sepsis-coded hospitalisation incidence and trends

**Fig. 3:**
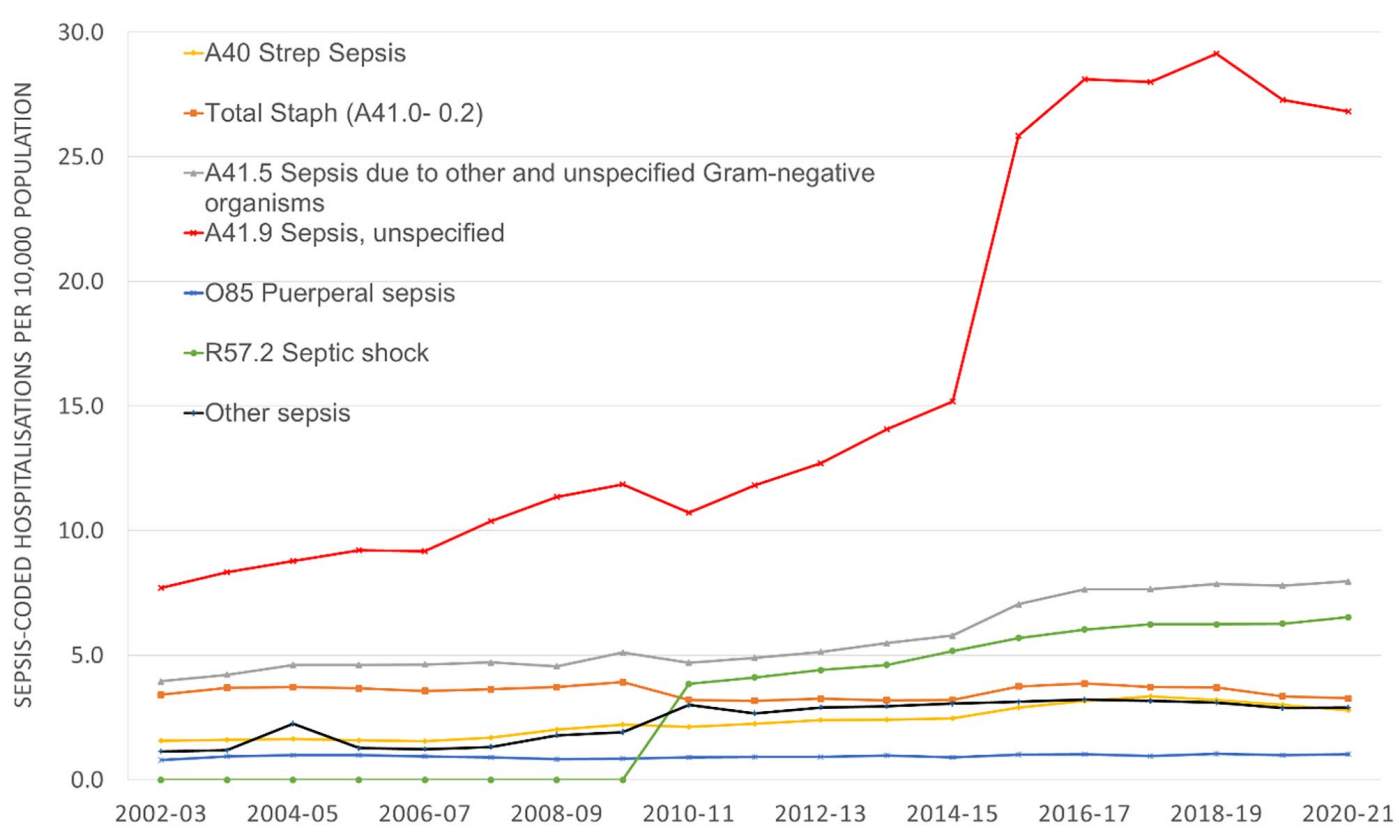
Incidence of sepsis-coded hospitalisations by ICD-10-AM codes. Note: Hospitalisations coded as other sepsis codes were calculated by adding hospitalisations coded as sepsis ICD-10-AM codes other than the top six sepsis ICD-10-AM codes displayed in the graph; ICD-10-AM: International Classification of Diseases Australian-modification. Code R57.2 for septic shock was introduced in 2009–2010

The population-based and hospital-based incidences of sepsis-coded hospitalisations increased from 18.6 to 51.3/10,000 population and 55.1 to 111.4/10,000 hospitalisations respectively during the study period. The average annual percentage increase for population-based and hospital-based sepsis incidence were 5.8% (95% CI: 2.9-8.7%) and 4.1% (95% CI: 1.0-7.3%), respectively with both being statistically significantly different from zero (p < 0.01) (Supplementary file 3; Figure S1). The two time points at which a notable change in the trend of sepsis-coded hospitalisations incidence was observed were 2012-13 and 2016-17. Population-based incidence of sepsis-coded hospitalisations for unspecified sepsis (A41.9) increased from 7.7 to 26.8 per 10,000 population during the study period whereas for other common sepsis codes such as sepsis due to unspecified gram-negative organisms (A41.5), it increased from 4.0 to 8.0 per 10,000 population and for septic shock (R57.2), from 3.8 to 6.5 per 10,000 population during the study period. (Fig. 3)

### Sepsis-coded hospitalisations and incidence in subgroups

Hospitalisations coded as sepsis and their population-based incidences in predefined subgroups are shown in Supplementary file 3; Table S2 and Figure S2. Sepsis-coded hospitalisations increased more in elderly patients (those age 65 and over) than in younger patients; difference in average annual percentage change 8.5% (95% CI: 4.8–12.3%), P < 0.01. Average annual percentage change was not different in males versus females (difference 1.4% (95% CI -2.3–5.1%), P = 0.46) or in elderly males versus elderly females (difference 2.8% (95% CI -1.6–7.3%), P = 0.21).

### Sepsis-coded hospitalisations associated costs (table 1)

In Australia the total annual cost associated with sepsis-coded hospitalisations increased from AUD199M (€128 M) in 2012 to AUD711M (€455 M) in 2019, an average annual percentage increase of 16.7% (95% CI: 5.5–29.2%) (p < 0.01). The number of hospitalisations assigned AR-DRG ‘Septicaemia with catastrophic outcomes’ until 2014-15, thereafter Septicaemia, Major Complexity (T60A) increased from 7598 to 12,974 during the financial years 2012-15. Introduction of a third sepsis AR-DRG ‘Septicaemia, Minor Complexity’ (T60C) in financial year 2015–2016 was associated with a reduction in the number of hospitalisations assigned to Septicaemia, Major Complexity (T60A) in that year which then consistently increased to 12,288 in 2018-19. The number of hospitalisations assigned AR-DRG ‘Septicaemia without catastrophic outcomes’ until 2015-16, thereafter Septicaemia, Intermediate Complexity’ (T60B) increased from 9578 in 2011-12 to 16,611 in 2018-19. The number of hospitalisations assigned Septicaemia, Minor Complexity’ (T60C) increased from 12,811 in 2015-16 (the year it was introduced) to 16,030 in 2018-19. Details of the cost weight, average cost per AR-DRG for sepsis-associated AR-DRGs and the Australian national efficient price are provided in Table 1.

**Table 1:** Sepsis AR-DRGs and associated cost in Australian dollars (Euro, €)

Year	National Efficient Price	T60A - Septicaemia, Major Complexity*				T60B - Septicaemia, Intermediate Complexity*				T60C - Septicaemia, Minor Complexity*				Total annual cost
		Cost weight	Average cost per AR-DRG	Number of AR-DRG	Associated cost	Cost weight	Average cost per AR-DRG	Number of AR-DRGs	Associated cost	Cost weight	Average cost per AR-DRG	Number of AR-DRGs	Associated cost	
2011-12	4,676	3.5	16,301	7,598	124 M	1.7	7,819	9,578	75 M					199 M (€128 M)
2012-13	4,808	3.1	15,409	9,115	140 M	1.6	7,732	9,802	76 M					216 M (€140 M)
2013-14	4,993	3.1	15,278	11,095	175 M	1.5	7,690	10,406	78 M					253 M (€164 M)
2014-15	5,007	4.7	24,575	12,974	319 M	2.5	12,694	10,575	134 M					453 M (€292 M)
2015-16	4,971	4.7	25,461	5,793	147 M	2.4	12,643	12,329	156 M	1.4	7,109	12,811	91	394 M (€255 M)
2016-17	4,883	4.8	24,937	10,261	256 M	2.2	11,530	16,637	170 M	1.3	6,651	18,538	123	549 M (€355 M)
2017-18	4,910	5.5	26,884	10,571	284 M	2.6	12,609	15,988	202 M	1.3	6,287	16,964	107	592 M (€283 M)
2018-19	5,012	5.8	29,040	12,288	357 M	2.9	14,306	16,611	238 M	1.4	7,240	16,030	116	711 M (€455 M)

*T60A = Septicaemia, Major Complexity was known as Septicaemia with catastrophic outcomes until 2015-16); *T60B = Septicaemia, Intermediate Complexity was known as Septicaemia without catastrophic outcomes until 2015-16; T60C = Septicaemia, Minor Complexity was introduced in year 2016-17; M: Million; AR-DRG: Australian Refined Diagnosis-Related Groups

Note: The National Efficient Price is determined by the Independent Health and Aged Care Pricing Authority (formerly Independent Hospital Pricing Authority) and is used to determine the national efficient cost for health care services provided by public hospitals. Cost per hospital episode is calculated by multiplying the National Efficient Price by an assigned cost weight

## Discussion

### Statement of key findings

We found a significant increase in sepsis-coded hospitalisations in Australia between 2002 and 2021 and associated costs between 2012 and 2019. Sepsis-coded hospitalisations increased with age, particularly in those aged 65 years and above. The majority of sepsis codes in sepsis-coded hospitalisations were secondary diagnosis codes, and coding for unspecified sepsis accounted for the majority of the increase, both these findings may reflect poor or inconsistent documentation of sepsis in clinical records.

The increase in sepsis-coded hospitalisations was particularly marked between 2015 and 17 which coincided with the revision of the Australian Coding Standard for sepsis, suggesting that at least some of the increase may be due to changes in coding practices possibly aligned with increased awareness of sepsis. Factors that may produce an increase in the true incidence of sepsis include an ageing population, more widespread use of immunosuppressive treatments and improved survival in other conditions such as vascular diseases and a variety of malignancies. The disproportionate increase in hospitalisations coded as unspecified sepsis could be either due to inconsistencies in clinical documentation or coders unsure of the documentation.

### Comparison with previous studies

Estimates of sepsis incidence using ICD coding use two broad approaches. The first approach is to count only episodes where a specific sepsis code is recorded, this is referred to as the *explicit* criteria [6]. The second approach is to count episodes where a code for infection is combined with a code that indicates organ dysfunction, referred to as *implicit* criteria as the presence of infection and organ dysfunction implies sepsis [6]. Both these methods have limitations. Notably for the *implicit* criteria the temporal and causal relationships between infection and organ dysfunction are assumed rather than proven. In a recent US study that used retrospective clinical diagnosis to examine the accuracy of these methods the explicit method undercounted sepsis while the implicit criteria significantly over counted sepsis [7].

Two other Australian studies have reported changes in sepsis-coded hospitalisations over shorter time periods [20, 21]. Both studies reported an increase in the number of sepsis-coded hospitalisations during their periods of study. The finding of most sepsis-coded hospitalisations being secondary diagnosis codes in our study is similar to the another study conducted for the Australian Commission of Safety and Quality in Healthcare [22]. Similar studies conducted in other countries during the same period reported widely varying incidences of coding of sepsis. Imeda et al. [23]. reported an increasing trend of sepsis in Japan with annual increase of 0.30% between 2010 and 17 which is much lower than our study (5.8%). Martin et al. [24] reported an annual increase of 8.7% in the sepsis incidence in the US between 1979 and 2000 which is higher than our study but was conducted prior to our study. In a later study of the Medicare benefited population in the US between 2012 and 2018, Buchman et al. reported similar results to ours with a trend of increasing sepsis hospitalisations and associated costs. Three studies reported sepsis trends in Spain, first [25] reported a lower sepsis incidence compared to our study (6.4 to 10.5 per 10,000 population during 2006-11), second [8] used a broader Angus criteria and wider duration and reported similar incidence (29 and 48 per 10,000 population in 2000 and 2013, respectively) while third study in young adults reported lower incidence ranging between 1.4 and 1.7 per 10,000 population between 2006 and 15 with an annual average percent change of 1.5% [26]. Lastly, a study conducted in Taiwan [27] reported a much higher sepsis incidence ranging from 62.3 to 64.7 per 10,000 population during 2010-14 but did not report an increasing incidence over time.

In Australia, a new sepsis AR-DRG of “septicaemia, minor complexity” was introduced in 2015-16 which was subsequently assigned to about 40% of the total sepsis related AR-DRGs. As it assigns the lowest cost, this resulted in marked reduction in estimated total cost of sepsis-coded hospitalisations in that year. In our study the annual cost of sepsis-coded hospitalisations in Australia was AUD711M (€455 M) in the financial year 2018-19 which is similar to the direct cost of sepsis estimated in another Australian report [13].

### Strengths & limitations

The strengths of our study include the use of a reliable prospectively collected national database consistently maintained over a 20-year period and the use of ICD codes modified from the Global Burden of Disease Study with the assistance of the lead author of that study [3]. However, our study has limitations including those inherent in using ICD coding to study sepsis epidemiology. Moreover, it is not possible to quantify the contributions of an actual increase in sepsis cases versus changes in coding practices and increased awareness about sepsis to the observed increase in sepsis-coded hospitalisations. Lastly, hospitalisation costs were not available for the entire study duration which means we could not compare the trends in sepsis-coded hospitalisations with the associated cost over the same periods.

### Implications for clinicians, researchers, and policymakers

Notwithstanding the limitations noted above, it is clear sepsis places considerable burden and costs on the healthcare system and community. Thus, training in the recognition and management of sepsis and to document sepsis accurately in medical records should be a high priority for clinicians. ICD coding is currently the only practical way to study sepsis epidemiology at scale and over time. Research to improve the accuracy of sepsis ICD coding would enable standardised and more robust case identification. The increasing incidence and cost associated with sepsis-coded hospitalisations emphasizes the need for policy makers to take the steps recommended in the WHO resolution to improve the prevention, recognition and management of sepsis and so to reduce its human and financial burden [2].

## Conclusion

In Australia, there has been a significant increase in sepsis-coded hospitalisations over the last 20 years and also, where data is available, in associated costs. The increase in incidence has occurred mainly in those aged 65 years and over, and predominantly due to an increase in coding of unspecified sepsis. Strengthening the use of ICD coding to study sepsis, as recommended by the WHO, should be a research and policy priority.

### Electronic supplementary material

Below is the link to the electronic supplementary material.

Supplementary Material 1

Supplementary Material 2

Supplementary Material 3

## Data Availability

The primary ICD coding and ARDRG data that support the findings of this study is publicly available at: https://www.aihw.gov.au/reports/hospitals/principal-diagnosis-data-cubes and https://www.aihw.gov.au/reports/hospitals/ar-drg-data-cubes/contents/data-cubes. Secondary diagnosis ICD coding data is not freely available as it was provided by AIHW for the purpose of this study.

## References

[1] Singer M, Deutschman CS, Seymour CW, Shankar-Hari M, Annane D, Bauer M (2016). The Third International Consensus definitions for Sepsis and septic shock (Sepsis-3). JAMA.

[2] Reinhart K, Daniels R, Kissoon N, Machado FR, Schachter RD, Finfer SJNEJoM (2017). Recognizing sepsis as a global health priority—a. WHO Resolution.

[3] Rudd KE, Johnson SC, Agesa KM, Shackelford KA, Tsoi D, Kievlan DR (2020). Global, regional, and national sepsis incidence and mortality, 1990–2017: analysis for the global burden of Disease Study. Lancet (London England).

[4] WHO. International Statistical Classification of Diseases and Related Health Problems (ICD) [Available from: https://www.who.int/standards/classifications/classification-of-diseases.

[5] Rhee C, Dantes R, Epstein L, Murphy DJ, Seymour CW, Iwashyna TJ (2017). Incidence and trends of sepsis in US hospitals using clinical vs claims data, 2009–2014. JAMA - Journal of the American Medical Association.

[6] Gobatto ALN, Besen BAMP, Azevedo LCPJS. How can we estimate sepsis incidence and, mortality?. 2017;47(1S):6–11. 10.1097/SHK.0000000000000703 27454379

[7] Rudd KE, Delaney A, Finfer S (2017). Counting Sepsis, an imprecise but improving science. JAMA.

[8] Álvaro-Meca A, Jiménez-Sousa MA, Micheloud D, Sánchez-Lopez A, Heredia-Rodríguez M, Tamayo E et al. Epidemiological trends of sepsis in the twenty-first century (2000–2013): an analysis of incidence, mortality, and associated costs in Spain. 2018;16(1):1–11. 10.1186/s12963-018-0160-x PMC5809921 29433513

[9] Kadri SS, Rhee C, Strich JR, Morales MK, Hohmann S, Menchaca J et al. Estimating ten-year trends in septic shock incidence and mortality in United States Academic Medical centers using Clinical Data2017. 10.1016/j.chest.2016.07.010 PMC5310115 27452768

[10] De La Suarez A, Gilsanz F, Maseda E (2016). Epidemiologic trends of sepsis in western countries. Ann Transl Med.

[11] Kaukonen K, Bailey M, Suzuki S, Pilcher D, Bellomo R (2014). Mortality related to severe sepsis and septic shock among critically ill patients in Australia and New Zealand, 2000–2012. JAMA.

[12] Lee C-C, Yo C-H, Lee M-tG, Tsai K-C, Lee S-H, Chen Y-S (2017). Adult sepsis–a nationwide study of trends and outcomes in a population of 23 million people. J Infect.

[13] Cost of sepsis in Australia report. The George Institute for Global Health; 2021.

[14] IHPA. Australian Coding Standards (ACS). 2020.

[15] Health, AIo (2019). Welfare. Principal diagnosis data cubes.

[16] AIHW. Australian refined diagnosis-related groups (AR-DRG) data cubes.

[17] Australia Bureau of Statistics. National, state and territory population. [Internet]. [cited May 2022]. Available from: https://www.abs.gov.au/statistics/people/population/national-state-and-territory-population/latest-release.

[18] IHPA. National Hospital Cost Data Collection.

[19] Thompson KJ, Finfer SR, Coombes J, Eades S, Hunter K, Leong RNF (2021). Incidence and outcomes of sepsis in Aboriginal and Torres Strait Islander and non-indigenous residents of New South Wales: Population-based cohort study. Crit Care Resusc.

[20] Ore T (2016). Trends and disparities in sepsis hospitalisations in Australia.pdf. Aust Health Rev.

[21] Sundararajan V, MacIsaac CM, Presneill JJ, Cade JF, Visvanathan KJC (2005). Epidemiology of sepsis in Victoria, Australia. J Crit care Med.

[22] Li LSN, Rathnayake K, Westbrook JI. Epidemiology of Sepsis in Australian Public Hospitals: A Mixed Methods, National Longitudinal Study (2013–2018) Australian Commission on Safety and Quality in Health Care 2020.

[23] Imaeda T, Nakada T-a, Takahashi N, Yamao Y, Nakagawa S, Ogura H (2021). Trends in the incidence and outcome of sepsis using data from a Japanese nationwide medical claims database-the Japan Sepsis Alliance (JaSA) study group. Crit Care.

[24] Martin GS, Mannino DM, Eaton S, Moss M (2003). The epidemiology of sepsis in the United States from 1979 through 2000. N Engl J Med.

[25] Bouza C, López-Cuadrado T, Saz-Parkinson Z, Amate-Blanco JM (2014). Epidemiology and recent trends of severe sepsis in Spain: a nationwide population-based analysis (2006–2011). BMC Infect Dis.

[26] Bouza C, Lopez-Cuadrado T. Epidemiology and trends of sepsis in young adults aged 20–44 years: a nationwide population-based study. J Clin Med. 2020;9 (1) (no pagination)(77). 10.3390/jcm9010077 PMC7019795 31892221

[27] Chen Y-J, Chen F-L, Chen J-H, Wu M-TM, Chen Y-L, Chien D-S et al. Epidemiol sepsis Taiwan. 2019;98(20). 10.1097/MD.0000000000015725 PMC6531136 31096527

