# Supplementary material for: Sepsis-coded hospitalisations and associated costs in Australia: a retrospective analysis
Source: BMC Health Serv Res. 2023 Nov 29;23:1319. doi: 10.1186/s12913-023-10223-1 (PMC10688047; doi:10.1186/s12913-023-10223-1)
Supplement: Supplementary file 1 — Supplementary Material 1 [file 12913_2023_10223_MOESM1_ESM.docx]

**Sepsis-related AR-DRGs**

| AR-DRG | Sepsis subtype |
| --- | --- |
| T60A | Septicaemia with Catastrophic complications and comorbidities until 2014-15, subsequently *Septicaemia, Major Complexity* |
| T60B | Septicaemia without catastrophic complications and comorbidities until 2014-15, subsequently *Septicaemia, Intermediate Complexity* |
| T60C | Septicaemia, Minor Complexity from 2015-16 onwards |

*AR-DRG: Australian Refined Diagnosis-Related Group*

*Note: A new AR-DRG classification system was introduced for sepsis in 2015-16 which replaced two previous sepsis-related AR-DRGs with three AR-DRGs (Major, intermediate and minor complexity)*
