# Supplementary material for: Sepsis-coded hospitalisations and associated costs in Australia: a retrospective analysis
Source: BMC Health Serv Res. 2023 Nov 29;23:1319. doi: 10.1186/s12913-023-10223-1 (PMC10688047; doi:10.1186/s12913-023-10223-1)
Supplement: Supplementary file 2 — Supplementary Material 2 [file 12913_2023_10223_MOESM2_ESM.docx]

**Modified GBD sepsis ICD-10-AM codes**

| **A02.1** | Salmonella sepsis |
| --- | --- |
| **A22.7** | Anthrax sepsis |
| **A24.1** | Acute and fulminating melioidosis |
| **A26.7** | Erysipelothrix sepsis |
| **A28.2** | Extraintestinal yersiniosis |
| **A32.7** | Listerial sepsis |
| **A39.4** | Meningococcaemia Unspecified |
| **A40** | Streptococcal Sepsis |
| **A41.0- 0.2** | Sepsis due to any staphylococcus |
| **A41.3** | Sepsis due to Haemophilus influenzae |
| **A41.4** | Sepsis due to anaerobes |
| **A41.5** | Sepsis due to other and unspecified Gram-negative organisms |
| **A41.8** | Other specified sepsis |
| **A41.9** | Sepsis, unspecified |
| **A42.7** | Actinomycotic sepsis |
| **A54.8** | Other gonococcal infections |
| **B00.7** | Disseminated Herpes viral disease |
| **B37.7** | Candidal sepsis |
| **O85** | Puerperal sepsis |
| **R57.2** | Septic shock |
| **R65.1** | Severe sepsis/SIRS with organ failure |

SIRS: Systemic Inflammatory Response Syndrome
