# Supplementary material for: Sepsis-coded hospitalisations and associated costs in Australia: a retrospective analysis
Source: BMC Health Serv Res. 2023 Nov 29;23:1319. doi: 10.1186/s12913-023-10223-1 (PMC10688047; doi:10.1186/s12913-023-10223-1)
Supplement: Supplementary file 3 — Supplementary Material 3 [file 12913_2023_10223_MOESM3_ESM.docx]

**Table S1 Number and proportion of hospitalisations coded as sepsis ICD-10-AM codes**

| ICD-10-AM codes | | 2002-03 | 2003-04 | 2004-05 | 2005-06 | 2006-07 | 2007-08 | 2008-09 | 2009-10 | 2010-11 | 2011-12 | 2012-13 | 2013-14 | 2014-15 | 2015-16 | 2016-17 | 2017-18 | 2018-19 | 2019-20 | 2020-21 |
| --- | --- | --- | --- | --- | --- | --- | --- | --- | --- | --- | --- | --- | --- | --- | --- | --- | --- | --- | --- | --- |
| Sepsis codes | Principal | 13843 | 14919 | 15378 | 15810 | 15988 | 17301 | 19081 | 21040 | 18954 | 21022 | 23002 | 26047 | 28221 | 38410 | 47727 | 45672 | 46914 | 45021 | 44186 |
|  | Additional | 22785 | 24899 | 26949 | 27816 | 27966 | 30832 | 33565 | 35967 | 44,747 | 46818 | 50343 | 53106 | 56978 | 81069 | 82838 | 86919 | 90702 | 87325 | 87640 |
|  | **Total** | **36628** | **39818** | **42327** | **43686** | **43954** | **48133** | **52646** | **57007** | **63701** | **67840** | **73345** | **79153** | **85199** | **119479** | **130565** | **132591** | **137616** | **132346** | **131826** |
| A41.9 | N | 15178 | 16609 | 17705 | 18850 | 19122 | 22058 | 24622 | 26124 | 23968 | 26866 | 29387 | 33042 | 36170 | 62542 | 69164 | 69932 | 73854 | 70024 | 68910 |
|  | *Proportion* | *41.44%* | *41.71%* | *39.89%* | *43.15%* | *43.50%* | *45.83%* | *46.77%* | *45.83%* | *37.63%* | *39.60%* | *40.07%* | *41.74%* | *42.45%* | *52.35%* | *52.97%* | *52.74%* | *53.67%* | *52.91%* | *52.27%* |
| A41.5 | N | 7809 | 8401 | 9302 | 9428 | 9648 | 10015 | 9893 | 11262 | 10510 | 11138 | 11867 | 12883 | 13788 | 17054 | 18806 | 19107 | 19915 | 19994 | 20466 |
|  | *Proportion* | *21.32%* | *21.10%* | *20.96%* | *21.58%* | *21.95%* | *20.81%* | *18.79%* | *19.76%* | *16.50%* | *16.42%* | *16.18%* | *16.28%* | *16.18%* | *14.27%* | *14.40%* | *14.41%* | *14.47%* | *15.11%* | *15.53%* |
| A41.0- 0.2 | N | 6737 | 7370 | 7520 | 7509 | 7433 | 7718 | 8069 | 8638 | 7166 | 7209 | 7535 | 7488 | 7635 | 9064 | 9508 | 9295 | 9397 | 8612 | 8408 |
|  | *Proportion* | *18.39%* | *18.51%* | *16.94%* | *17.19%* | *16.91%* | *16.03%* | *15.33%* | *15.15%* | *11.25%* | *10.63%* | *10.27%* | *9.46%* | *8.96%* | *7.59%* | *7.28%* | *7.01%* | *6.83%* | *6.51%* | *6.38%* |
| A40 | N | 3085 | 3200 | 3304 | 3259 | 3227 | 3617 | 4363 | 4881 | 4755 | 5122 | 5525 | 5676 | 5861 | 7027 | 7793 | 8375 | 8126 | 7695 | 7167 |
|  | *Proportion* | *8.42%* | *8.04%* | *7.44%* | *7.46%* | *7.34%* | *7.51%* | *8.29%* | *8.56%* | *7.46%* | *7.55%* | *7.53%* | *7.17%* | *6.88%* | *5.88%* | *5.97%* | *6.32%* | *5.90%* | *5.81%* | *5.44%* |
| O85 | N | 1566 | 1872 | 1995 | 2030 | 1959 | 1917 | 1819 | 1882 | 2011 | 2094 | 2119 | 2305 | 2148 | 2438 | 2535 | 2402 | 2647 | 2573 | 2632 |
|  | *Proportion* | *4.28%* | *4.70%* | *4.50%* | *4.65%* | *4.46%* | *3.98%* | *3.46%* | *3.30%* | *3.16%* | *3.09%* | *2.89%* | *2.91%* | *2.52%* | *2.04%* | *1.94%* | *1.81%* | *1.92%* | *1.94%* | *2.00%* |
| A41.8 | N | 991 | 1068 | 1197 | 1177 | 1224 | 1393 | 2612 | 2906 | 2712 | 2588 | 2889 | 3061 | 3270 | 4335 | 4767 | 4819 | 4927 | 4911 | 5015 |
|  | *Proportion* | *2.71%* | *2.68%* | *2.70%* | *2.69%* | *2.78%* | *2.89%* | *4.96%* | *5.10%* | *4.26%* | *3.81%* | *3.94%* | *3.87%* | *3.84%* | *3.63%* | *3.65%* | *3.63%* | *3.58%* | *3.71%* | *3.80%* |
| A39.4 | N | 272 | 223 | 2185 | 179 | 99 | 124 | 110 | 86 | 97 | 99 | 88 | 76 | 89 | 88 | 163 | 177 | 154 | 99 | 39 |
|  | *Proportion* | *0.74%* | *0.56%* | *4.92%* | *0.41%* | *0.23%* | *0.26%* | *0.21%* | *0.15%* | *0.15%* | *0.15%* | *0.12%* | *0.10%* | *0.10%* | *0.07%* | *0.12%* | *0.13%* | *0.11%* | *0.07%* | *0.03%* |
| B37.7 | N | 376 | 387 | 414 | 470 | 551 | 514 | 662 | 696 | 598 | 641 | 622 | 550 | 649 | 707 | 790 | 812 | 724 | 713 | 762 |
|  | *Proportion* | *1.03%* | *0.97%* | *0.93%* | *1.08%* | *1.25%* | *1.07%* | *1.26%* | *1.22%* | *0.94%* | *0.94%* | *0.85%* | *0.69%* | *0.76%* | *0.59%* | *0.61%* | *0.61%* | *0.53%* | *0.54%* | *0.58%* |
| A41.3 | N | 77 | 111 | 92 | 104 | 92 | 88 | 118 | 108 | 123 | 113 | 119 | 101 | 144 | 178 | 197 | 158 | 186 | 160 | 111 |
|  | *Proportion* | *0.21%* | *0.28%* | *0.21%* | *0.24%* | *0.21%* | *0.18%* | *0.22%* | *0.19%* | *0.19%* | *0.17%* | *0.16%* | *0.13%* | *0.17%* | *0.15%* | *0.15%* | *0.12%* | *0.14%* | *0.12%* | *0.08%* |
| A41.4 | N | 293 | 282 | 358 | 372 | 304 | 388 | 141 | 164 | 177 | 223 | 236 | 219 | 248 | 352 | 362 | 456 | 487 | 482 | 538 |
|  | *Proportion* | *0.80%* | *0.71%* | *0.81%* | *0.85%* | *0.69%* | *0.81%* | *0.27%* | *0.29%* | *0.28%* | *0.33%* | *0.32%* | *0.28%* | *0.29%* | *0.29%* | *0.28%* | *0.34%* | *0.35%* | *0.36%* | *0.41%* |
| A02.1 | N | 76 | 90 | 86 | 89 | 101 | 94 | 99 | 129 | 125 | 121 | 124 | 138 | 142 | 167 | 203 | 169 | 150 | 134 | 109 |
|  | *Proportion* | *0.21%* | *0.23%* | *0.19%* | *0.20%* | *0.23%* | *0.20%* | *0.19%* | *0.23%* | *0.20%* | *0.18%* | *0.17%* | *0.17%* | *0.17%* | *0.14%* | *0.16%* | *0.13%* | *0.11%* | *0.10%* | *0.08%* |
| A32.7 | N | 32 | 32 | 38 | 30 | 25 | 47 | 31 | 53 | 47 | 44 | 48 | 41 | 43 | 31 | 35 | 36 | 25 | 16 | 7 |
|  | *Proportion* | *0.09%* | *0.08%* | *0.09%* | *0.07%* | *0.06%* | *0.10%* | *0.06%* | *0.09%* | *0.07%* | *0.06%* | *0.07%* | *0.05%* | *0.05%* | *0.03%* | *0.03%* | *0.03%* | *0.02%* | *0.01%* | *0.01%* |
| B00.7 | N | 13 | 14 | 19 | 25 | 18 | 19 | 17 | 16 | 20 | 23 | 29 | 29 | 37 | 34 | 43 | 18 | 29 | 40 | 54 |
|  | *Proportion* | *0.04%* | *0.04%* | *0.04%* | *0.06%* | *0.04%* | *0.04%* | *0.03%* | *0.03%* | *0.03%* | *0.03%* | *0.04%* | *0.04%* | *0.04%* | *0.03%* | *0.03%* | *0.01%* | *0.02%* | *0.03%* | *0.04%* |
| A24.1 | N | 30 | 79 | 63 | 100 | 69 | 65 | 12 | 13 | 126 | 19 | 20 | 9 | 26 | 24 | 32 | 102 | 109 | 21 | 127 |
|  | *Proportion* | *0.08%* | *0.20%* | *0.14%* | *0.23%* | *0.16%* | *0.14%* | *0.02%* | *0.02%* | *0.20%* | *0.03%* | *0.03%* | *0.01%* | *0.03%* | *0.02%* | *0.02%* | *0.08%* | *0.08%* | *0.02%* | *0.10%* |
| A54.86 | N | 81 | 74 | 97 | 58 | 75 | 67 | 72 | 44 | 54 | 70 | 70 | 54 | 48 | 73 | 92 | 76 | 80 | 80 | 82 |
|  | *Proportion* | *0.22%* | *0.19%* | *0.22%* | *0.13%* | *0.17%* | *0.14%* | *0.14%* | *0.08%* | *0.08%* | *0.10%* | *0.10%* | *0.07%* | *0.06%* | *0.06%* | *0.07%* | *0.06%* | *0.06%* | *0.06%* | *0.06%* |
| A42.7 | N | 6 | 3 | 3 | 5 | 6 | 5 | 2 | 1 | 2 | 1 | 2 | 13 | 8 | 16 | 13 | 17 | 17 | 19 | 26 |
|  | *Proportion* | *0.02%* | *0.01%* | *0.01%* | *0.01%* | *0.01%* | *0.01%* | *0.00%* | *0.00%* | *0.00%* | *0.00%* | *0.00%* | *0.02%* | *0.01%* | *0.01%* | *0.01%* | *0.01%* | *0.01%* | *0.01%* | *0.02%* |
| R57.2 | N | NR | NR | NR | NR | NR | NR | NR | NR | 8589 | 9333 | 10186 | 10820 | 12323 | 13778 | 14831 | 15576 | 15810 | 16072 | 16777 |
|  | *Proportion* | - | - | - | - | - | - | - | - | *13.48%* | *13.76%* | *13.89%* | *13.67%* | *14.46%* | *11.53%* | *11.36%* | *11.75%* | *11.49%* | *12.14%* | *12.73%* |
| R65.1 | N | NR | NR | NR | NR | NR | NR | NR | NR | 2620 | 2136 | 2477 | 2644 | 2568 | 1570 | 1227 | 1061 | 976 | 694 | 594 |
|  | *Proportion* | - | - | - | - | - | - | - | - | *4.11%* | *3.15%* | *3.38%* | *3.34%* | *3.01%* | *1.31%* | *0.94%* | *0.80%* | *0.71%* | *0.52%* | *0.45%* |
| A28.2 | N | 4 | 3 | 1 | 1 | NR | 3 | 4 | 2 | 1 | NR | 1 | 1 | 1 | 1 | 2 | 1 | NR | NR | NR |
|  | *Proportion* | *0.01%* | *0.01%* | *0.00%* | *0.00%* | *-* | *0.01%* | *0.01%* | *0.00%* | *0.00%* | *-* | *0.00%* | *0.00%* | *0.00%* | *0.00%* | *0.00%* | *0.00%* | - | - | - |

ICD-10-AM: International Classification of Diseases 10^th^ Revision Australian Modification; NR: Not reported; SIRS: Systemic Inflammatory Response Syndrome

Note: Septicaemic plague (A20.7), Anthrax sepsis (A22.7), Generalized Tularaemia, (A21.7), and Erysipelpthrix sepsis (A26.7) were not reported in any year during the study period.

Note: Refer Supplementary file 2 for respective names of each sepsis ICD code.

**Table S2 Sepsis-coded hospitalisations and incidence in subgroups**

|  |  | 2002-03 | 2003-04 | 2004-05 | 2005-06 | 2006-07 | 2007-08 | 2008-09 | 2009-10 | 2010-11 | 2011-12 | 2012-13 | 2013-14 | 2014-15 | 2015-16 | 2016-17 | 2017-18 | 2018-19 | 2019-20 | 2020-21 |
| --- | --- | --- | --- | --- | --- | --- | --- | --- | --- | --- | --- | --- | --- | --- | --- | --- | --- | --- | --- | --- |
| Males | Primary | 7139 | 7594 | 7821 | 7927 | 8000 | 8817 | 9,777 | 10,785 | 9696 | 10774 | 11983 | 13606 | 14788 | 20551 | 25386 | 24405 | 25000 | 24274 | 23713 |
|  | Secondary | 12353 | 13417 | 14771 | 15248 | 15344 | 17112 | 18,740 | 20,219 | 24777 | 25427 | 27551 | 29448 | 31281 | 44765 | 45857 | 48019 | 50089 | 48283 | 48309 |
|  | Total | 19492 | 21011 | 22592 | 23175 | 23344 | 25929 | 28517 | 31004 | 34473 | 36201 | 39534 | 43054 | 46069 | 65316 | 71243 | 72424 | 75089 | 72557 | 72022 |
|  | *Incidence* | *19.9* | *21.2* | *22.5* | *22.8* | *22.5* | *24.5* | *26.4* | *28.3* | *31.0* | *32.0* | *34.4* | *36.9* | *39.0* | *54.4* | *58.4* | *58.4* | *59.7* | *57.0* | *56.5* |
| Females | Primary | 6687 | 7297 | 7507 | 7816 | 7958 | 8399 | 9,280 | 10,244 | 8916 | 10211 | 10976 | 12441 | 13433 | 17840 | 22321 | 21177 | 21927 | 20747 | 20473 |
|  | Secondary | 10432 | 11479 | 12177 | 12567 | 12622 | 13720 | 14,825 | 15,748 | 19970 | 21319 | 22792 | 23658 | 25697 | 36304 | 36981 | 38899 | 40613 | 39042 | 39330 |
|  | Total | 17119 | 18776 | 19684 | 20383 | 20580 | 22119 | 24105 | 25992 | 28886 | 31530 | 33768 | 36099 | 39130 | 54144 | 59302 | 60076 | 62540 | 59789 | 59803 |
|  | *Incidence* | *17.2* | *18.7* | *19.4* | *19.8* | *19.6* | *20.7* | *22.1* | *23.5* | *25.7* | *27.6* | *29.1* | *30.6* | *32.6* | *44.4* | *47.9* | *47.8* | *49.0* | *46.3* | *46.2* |
| Elderly Males | Primary | 4001 | 4367 | 4551 | 4856 | 4822 | 5473 | 6,085 | 6,805 | 6081 | 6954 | 7985 | 8942 | 9959 | 14074 | 17634 | 17274 | 17886 | 17559 | 17360 |
|  | Secondary | 7337 | 8026 | 8696 | 9063 | 9095 | 10260 | 11,208 | 12,021 | 15112 | 15900 | 17328 | 18565 | 19854 | 28842 | 29910 | 31375 | 33297 | 31813 | 32426 |
|  | Total | 11338 | 12393 | 13247 | 13919 | 13917 | 15733 | 17293 | 18826 | 21193 | 22854 | 25313 | 27507 | 29813 | 42916 | 47544 | 48649 | 51183 | 49372 | 49786 |
|  | *Incidence* | *73.3* | *79.3* | *83.7* | *86.7* | *85.1* | *94.2* | *101.3* | *108.6* | *120.6* | *127.9* | *139.2* | *149.2* | *159.5* | *226.3* | *246.6* | *248.5* | *257.5* | *245.5* | *247.1* |
| Elderly Females | Primary | 3500 | 3763 | 3919 | 4184 | 4363 | 4548 | 5,114 | 5,756 | 4929 | 5799 | 6325 | 7319 | 7999 | 10806 | 13,843 | 13103 | 13840 | 13106 | 13053 |
|  | Secondary | 5735 | 6439 | 6720 | 6849 | 6979 | 7805 | 8,611 | 8,956 | 11231 | 12139 | 13074 | 13419 | 14923 | 21182 | 21,749 | 22773 | 23944 | 22897 | 23296 |
|  | Total | 9235 | 10202 | 10639 | 11033 | 11342 | 12353 | 13725 | 14712 | 16160 | 17938 | 19399 | 20738 | 22922 | 31988 | 35592 | 35876 | 37784 | 36003 | 36349 |
|  | Incidence | 52.3 | 57.2 | 58.9 | 60.3 | 60.9 | 65.1 | 70.9 | 74.8 | 81.0 | 88.4 | 93.9 | 98.8 | 107.6 | 147.7 | 161.7 | 160.5 | 166.6 | 156.8 | 158.1 |
| Elderly (>= 65 years) | Primary | 7,501 | 8,130 | 8,470 | 9,040 | 9,185 | 10,021 | 11,199 | 12,561 | 11,010 | 12,753 | 14,310 | 16,261 | 17,958 | 24,880 | 31,477 | 30,377 | 31726 | 30665 | 30413 |
|  | Secondary | 13,072 | 14,465 | 15,416 | 15,912 | 16,074 | 18,065 | 19,819 | 20,977 | 26,343 | 28,039 | 30,402 | 31,984 | 34,777 | 50,024 | 51,659 | 54,148 | 57,241 | 54,710 | 55,722 |
|  | Total | 20573 | 22595 | 23886 | 24952 | 25259 | 28086 | 31018 | 33538 | 37353 | 40792 | 44712 | 48245 | 52735 | 74904 | 83136 | 84525 | 88967 | 85375 | 86135 |
|  | *Incidence* | *62.1* | *67.5* | *70.5* | *72.6* | *72.2* | *78.7* | *85.1* | *90.6* | *99.5* | *106.8* | *115.1* | *122.3* | *131.8* | *184.3* | *201.1* | *201.4* | *208.8* | *198.1* | *199.6* |
| Non-Elderly | Primary | 6691 | 6789 | 6908 | 6770 | 6803 | 7280 | 7882 | 8479 | 7944 | 8269 | 8692 | 9786 | 10263 | 13530 | 16250 | 15214 | 15201 | 14356 | 13773 |
|  | Secondary | 9713 | 10431 | 11532 | 11903 | 11892 | 12767 | 13746 | 14990 | 18404 | 18707 | 19941 | 21122 | 22201 | 31045 | 31179 | 32770 | 33461 | 32615 | 31917 |
|  | Total | 16404 | 17220 | 18440 | 18673 | 18695 | 20047 | 21628 | 23469 | 26348 | 26976 | 28633 | 30908 | 32464 | 44575 | 47429 | 47984 | 48662 | 46971 | 45690 |
|  | *Incidence* | *10.0* | *10.4* | *11.0* | *11.0* | *10.8* | *11.3* | *12.0* | *12.8* | *14.2* | *14.3* | *14.9* | *15.8* | *16.4* | *22.1* | *23.2* | *23.1* | *23.1* | *22.0* | *21.4* |

Note: Incidence is reported as per 10,000 population. Estimated resident population from June in a given year was used to calculate the incidence.

**Figure S1 Trend analysis of sepsis-coded hospitalisations incidence**


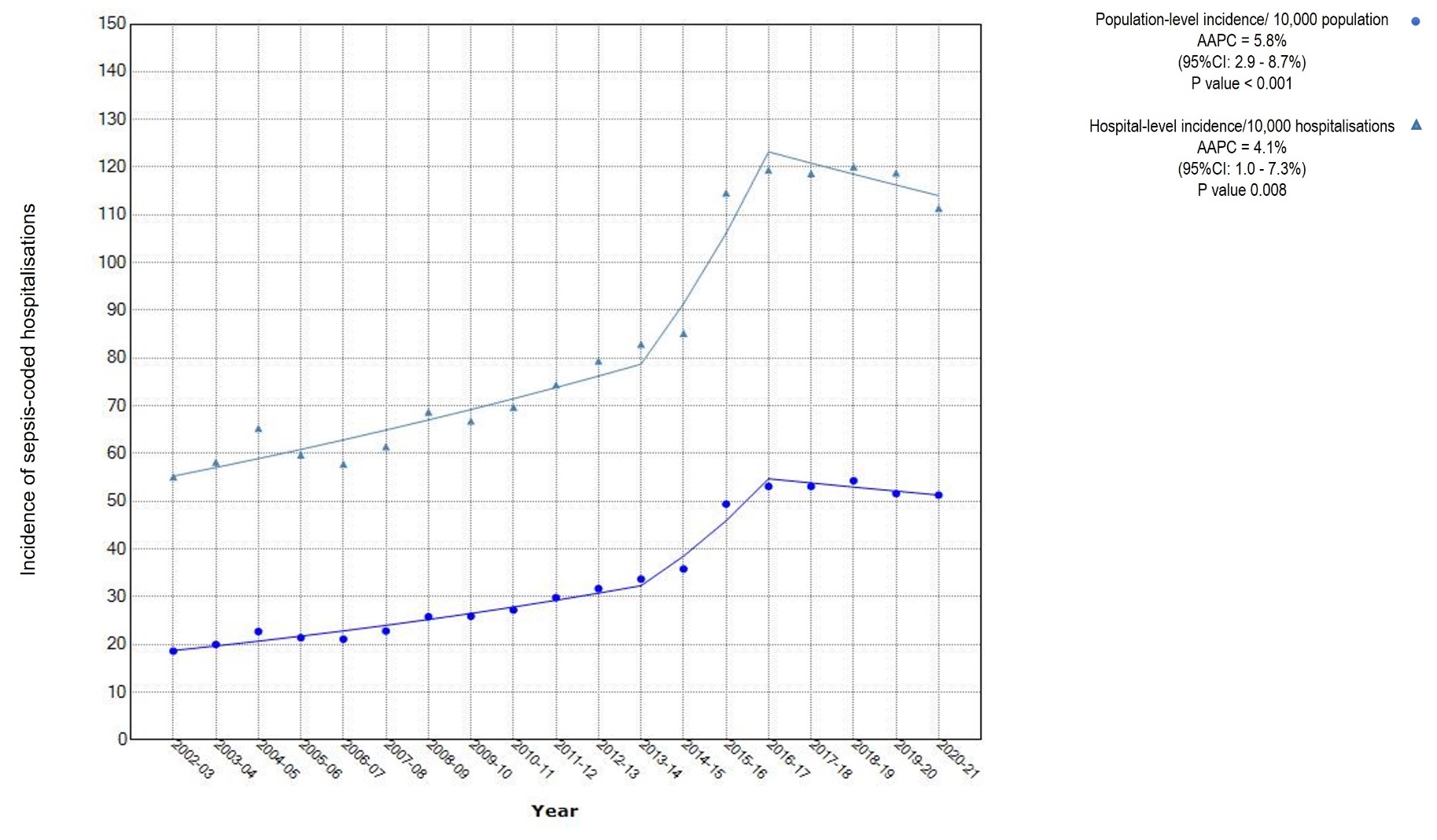


AAPC: Average Annual Percentage Change; CI: Confidence Interval

Note: Estimated resident population in Australia from June in a given year was used to calculate the population-level incidence.

**Figure S2 Annual percentage increase in sepsis-coded hospitalisations in subgroups**


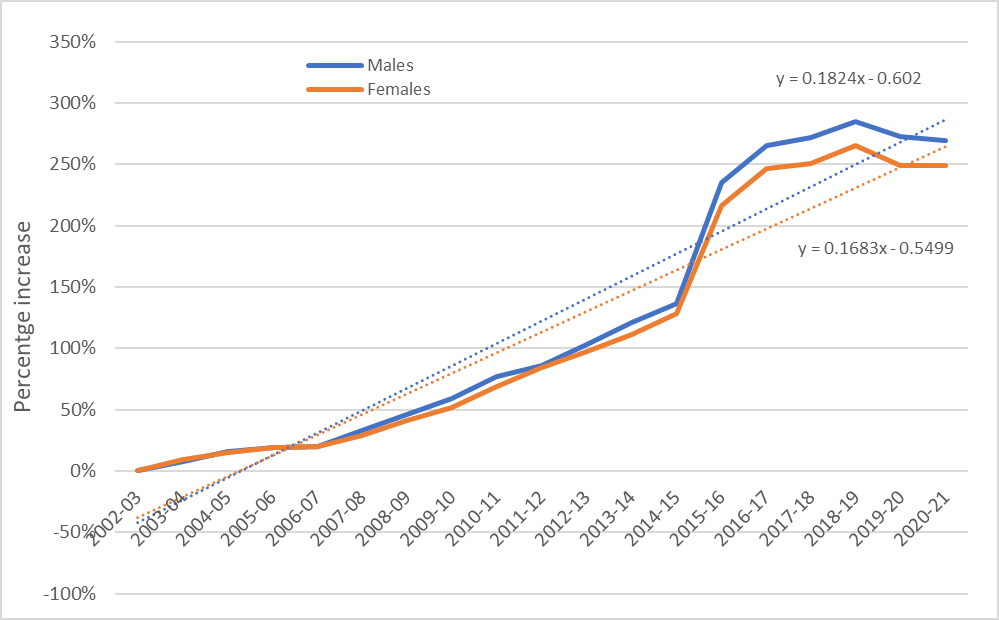

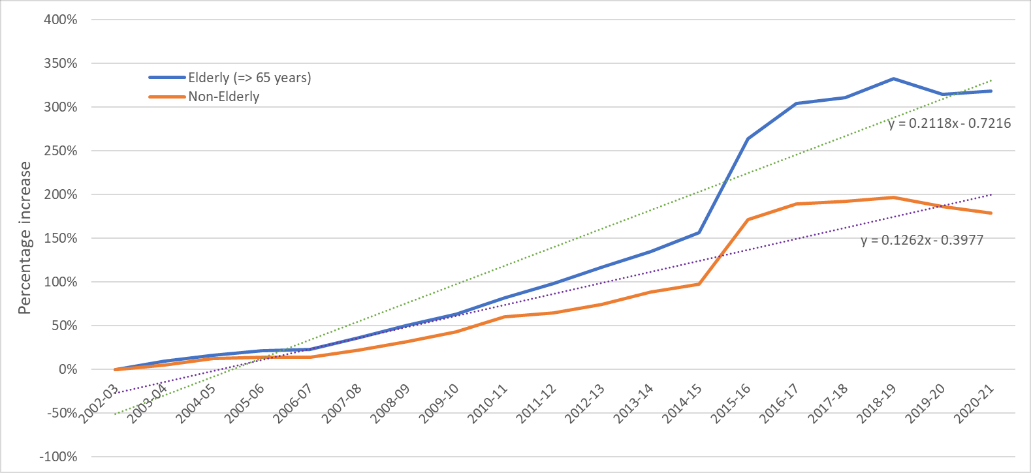

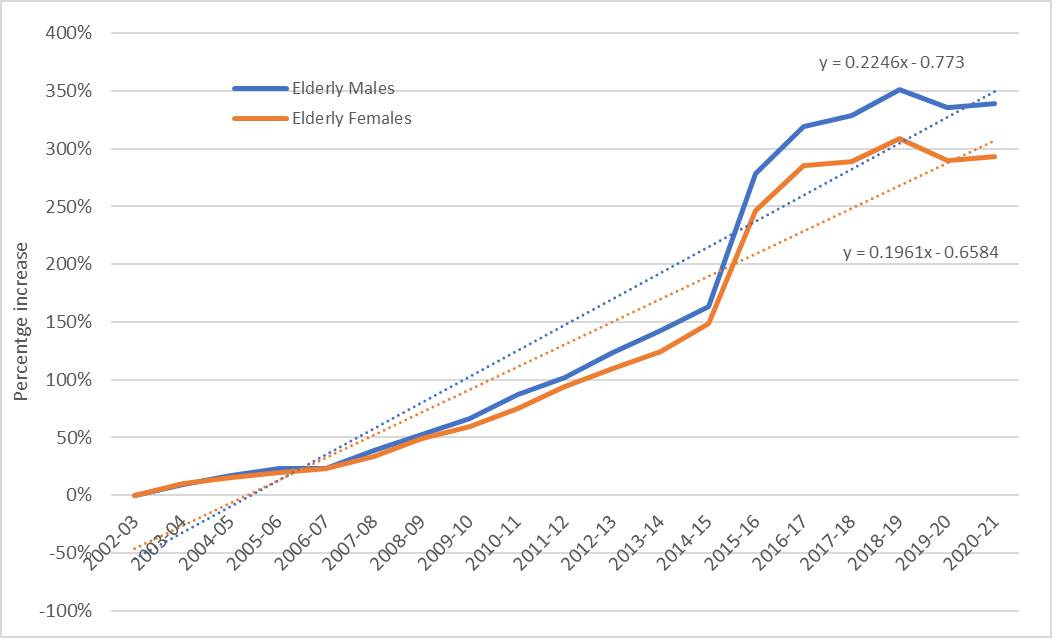


**S2a.** Elderly (>65 years) versus non-elderly

**S2c.** Elderly (>65 years) males versus females

**S2b.** Males versus females

Note: Total sepsis-coded hospitalisations were calculated by adding hospitalisations coded as individual sepsis ICD-10-AM codes as listed in Supplementary file 2.
